# Using RNA-Seq Data to Evaluate Reference Genes Suitable for Gene Expression Studies in Soybean

**DOI:** 10.1371/journal.pone.0136343

**Published:** 2015-09-08

**Authors:** Aldrin Kay-Yuen Yim, Johanna Wing-Hang Wong, Yee-Shan Ku, Hao Qin, Ting-Fung Chan, Hon-Ming Lam

**Affiliations:** 1 School of Life Sciences and Center for Soybean Research of the Partner State Key Laboratory of Agrobiotechnology, The Chinese University of Hong Kong, Shatin, Hong Kong, SAR, China; 2 Hong Kong Bioinformatics Centre, The Chinese University of Hong Kong, Shatin, Hong Kong, SAR, China; ISA, PORTUGAL

## Abstract

Differential gene expression profiles often provide important clues for gene functions. While reverse transcription quantitative real-time polymerase chain reaction (RT-qPCR) is an important tool, the validity of the results depends heavily on the choice of proper reference genes. In this study, we employed new and published RNA-sequencing (RNA-Seq) datasets (26 sequencing libraries in total) to evaluate reference genes reported in previous soybean studies. *In silico* PCR showed that 13 out of 37 previously reported primer sets have multiple targets, and 4 of them have amplicons with different sizes. Using a probabilistic approach, we identified new and improved candidate reference genes. We further performed 2 validation tests (with 26 RNA samples) on 8 commonly used reference genes and 7 newly identified candidates, using RT-qPCR. In general, the new candidate reference genes exhibited more stable expression levels under the tested experimental conditions. The three newly identified candidate reference genes *Bic-C2*, *F-box protein2*, and *VPS-like* gave the best overall performance, together with the commonly used *ELF1b*. It is expected that the proposed probabilistic model could serve as an important tool to identify stable reference genes when more soybean RNA-Seq data from different growth stages and treatments are used.

## Introduction

Expression of a gene may be tied to its involvement in a particular developmental stage, a response to environmental signals, and other cellular functions [1]. Tracking the changes in the expression level is therefore an important initial step to study the possible functions of a gene. To compare the expression of a target gene in different samples, the expression should first be normalized to a common factor. In the traditional northern blot analysis, the total RNA content is routinely used as the basis for normalization, with the assumption that its amount will remain more or less constant. On the other hand, reverse transcription coupled with real-time quantitative polymerase chain reaction (RT-qPCR) offers a more sensitive way to measure gene expression, which is also an important tool in plant research [2]. For a quantitative comparison of gene expressions using RT-qPCR, one critical factor is to choose suitable reference genes to normalize the expressions of target genes under different conditions [3–7]. Initially, the choice of reference genes was primarily based on the notion of “housekeeping” genes [4]. It is expected that since housekeeping genes encode proteins for essential cellular functions, they should be expressed at relatively stable levels in all cell or tissue types under various conditions. However, it is eventually known that many housekeeping genes do not express uniformly across different experimental settings [4, 6], and hence the choice of proper reference genes remains an important issue in gene expression analyses.

Within the plant research community, there is a strong call for the systematic validation of reference genes used in RT-qPCR [5]. For instance, many classical housekeeping genes in soybean exhibit variable levels of expression in different samples [8]. Due to the advent of next-generation sequencing technology, it is now possible to use RNA-Seq [9] data to empirically evaluate and identify stably expressed reference genes, disregarding the exact cellular functions of these genes. This approach is unbiased, analytically robust, specific to the model system of interest, and hence can provide a wider dynamic range for gene expression studies [4].

In this research, we made use of the deep-sequencing-based transcriptome data that we generated (6 datasets), together with transcriptome data available in public domains (20 datasets), to evaluate commonly-used reference genes in soybean research and identify new and more appropriate candidates.

## Results

### Evaluation of previously reported soybean reference genes using RNA-Seq data

To evaluate the stability of known reference genes commonly used in soybean research, we reviewed 8 previous studies that had evaluated and identified reference genes specifically for soybean [8, 10–16]. A total of 37 primer sets (from 28 reference genes) were retrieved. Since the genome annotation of *Glycine max* William 82 has been updated to v2.0, both the genomic sequences and the locus tags corresponding to the amplicons of these primer sets have been changed. *In silico* PCR was therefore performed to predict the amplicons of these primer sets and the results are shown in S1 Table. A total of 13 out of 37 primer sets might target amplicons corresponding to multiple gene loci. Although the coding sequences of these gene loci are mostly conserved, amplicons of different sizes were also revealed in four cases. For example, primer set for *TUA5* [8, 13, 15] is associated with 2 gene loci, Glyma.05G157300 and Glyma.08G115100, and at the locus Glyma.08G115100, this primer set will give two amplicons of 103bp and 129bp, respectively. Similarly, the primer set for *ELF1a* [10, 12, 15, 16] was predicted to give 2 amplicons of different sizes (161bp and 1056bp) from the gene locus Glyma.19G052400. Moreover, amplicons could not be identified based on *in silico* PCR for the primer sets of *SUBI2* and *PEPKR1/CDPK*. This may due to the latest update of the genome.

To quantify and examine the expression stability of reported reference genes, as well as to identify new candidate reference genes, 20 RNA-Seq libraries [17, 18] were downloaded from the websites of SoyKB [19, 20] and Phytozome [21]. In addition, 6 RNA deep-sequencing experiments were performed for the root, trifoliate, and primary leaves of young seedlings of one wild and one cultivated soybean accessions. All 26 libraries were mapped to the genome of *Glycine max* William 82 (S2 Table). The percentage of uniquely aligned reads varied among samples, ranging from 35% to 92%. The differences may be due to the sequencing parameters—paired-end versus single-end sequencing, sequence read lengths, and sequence read quality. The data from our 6 sequencing libraries exhibited the highest percentage of uniquely aligned reads, ranging from 87% to 92%, followed by the sequencing libraries by Schmutz et al. [17], with uniquely aligned reads ranging from 75% to 79%. Only 50% of the reads could be uniquely aligned to the genome when the sequencing data from Severin et al. [18] were used. Such relatively low percentage of uniquely aligned reads in the sequencing data may be related to the nature of the data—single-end sequencing and a shorter read length (35bp). Given the quality differences across 3 experiments, data from the 26 libraries were divided into two groups—Group 1 (our 6 sequencing libraries) and Group 2 (20 publicly available sequencing libraries). A novel probabilistic model was used for the normalization of gene expression across sequencing libraries, and a total of 25,036 genes were found to be commonly expressed in all 26 libraries.

**Fig 1:**
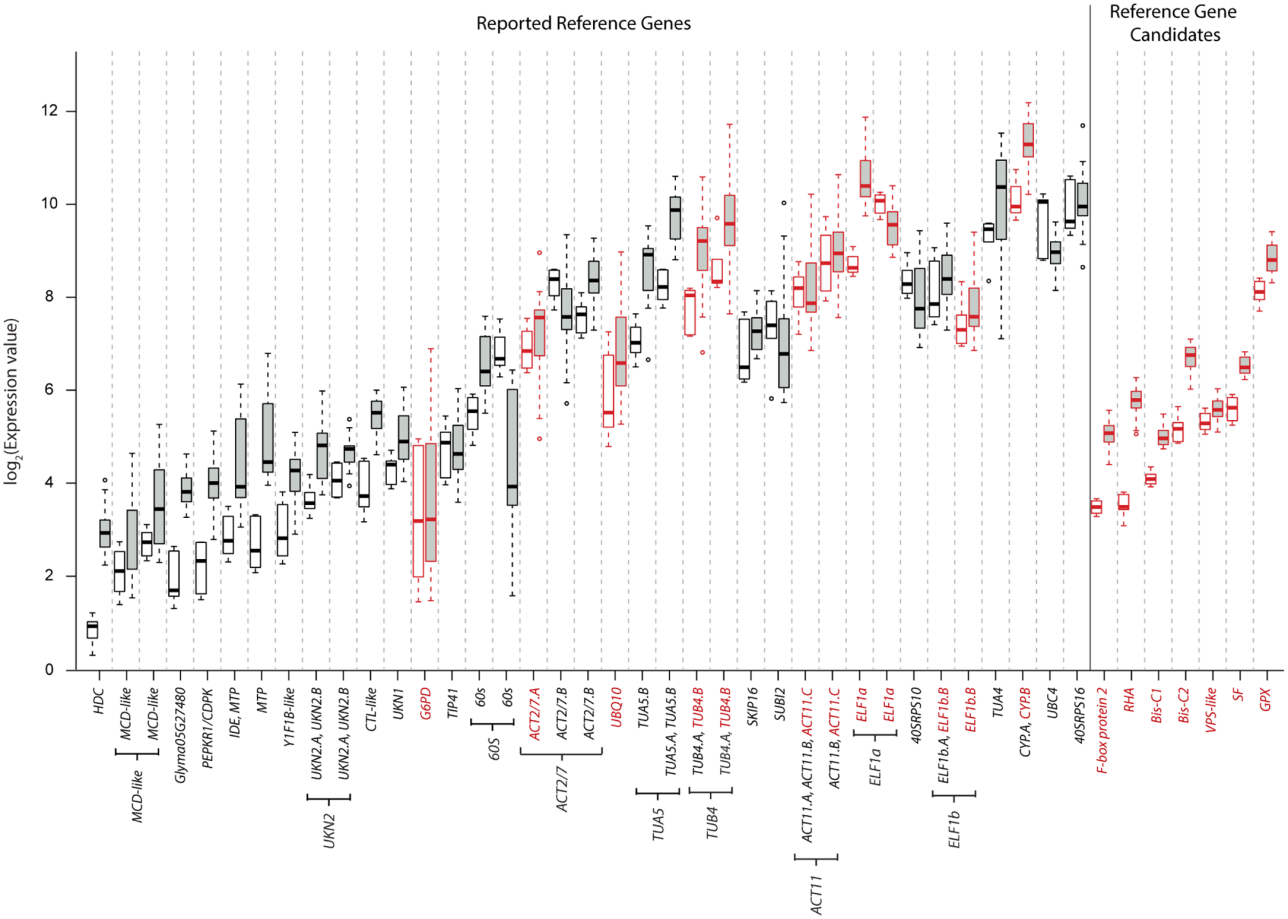
Evaluation of previously reported reference genes and new candidate reference genes. A boxplot of RNA-Seq data showing the expression levels of 37 previously reported primer sets from 28 known reference genes and 7 new candidate reference genes. The expression level of each target was shown. White boxes: Group 1 RNA-Seq data; Grey boxes: Group 2 RNA-Seq data. Primer sets with multiple targets were marked with brackets. Primer sets marked in red were chosen for RT-qPCR validation.

Fig 1 shows the expression levels of the targets corresponding to the 37 reported reference gene primer sets. Multiple targets from the same primer set were marked with a bracket. The expression levels of each gene in Group 1 and Group 2 sequencing libraries were shown in white and grey boxes, respectively. The *APC-like* gene exhibited extremely low read counts in all RNA-Seq data and hence was excluded in subsequent studies. In general, there was a higher degree of variation in Group 2 when compared to Group 1 for most targets. This is expected since Group 2 contains more sequencing libraries from different samples, as well as greater fluctuations in the sequencing and mapping quality. For each of the primer sets tested, the relative gene expression levels and degrees of variation in Group 1 and Group 2 showed similar trends. The performance of some reported reference genes was exceptionally poor, such as *G6PD* and *UBQ10*. Although the primer sets for *ELF1a*, *MCD-like*, *TUA5* and *UBC4* give stable expression patterns, the presence of multiple targets with different amplicon sizes is another issue of concern.

### Identification of new and improved candidate reference genes using RNA-Seq data

To identify new candidate reference genes, additional statistical analyses were performed. We considered two major factors: (i) Coefficient of Variation (CoV) of the gene; and (ii) p-value of two normality tests—Shapiro-Wilk normality test (Shapiro test) and Kolmogolov-Smirnov test (KS test). While the first factor reports the level of variations in gene expressions among samples, the second factor makes the assumption that for reference genes, the level of variations in gene expressions among samples should follow the Gaussian distribution (the null hypothesis). Using the two-stage selection process described in Materials and Method, 136 genes were selected out of 25,036 genes commonly expressed in 26 RNA-Seq libraries. The CoV and p-value of two normality tests for these 136 genes in both Group 1 and Group 2 were shown in S3 Table. We further selected 7 genes that have different expression levels–*F-box protein 2*, *RHA*, *Bic-C1*, *Bic-C2*, *VPS-like*, *SF* and *GPX* for experimental validation. All of these candidate reference genes have a low CoV and high p-value in the normality tests. The high p-value indicates that their expression variations followed a normal distribution and therefore the fluctuations in gene expression should not be sample-specific.

To cross-compare the stability of known reference genes commonly used in soybean gene expression studies (S1 Table) with the 7 candidate reference genes, we also selected 8 previously reported primer sets–*ACT2/7*.*A*, *ACT11*.*C*, *CYP*.*B*, *ELF1a*, *ELF1b*.*B*, *G6PD*, *TUB4*.*B*, and *UBQ10* for experimental validation (Table 1). The expression patterns of the 7 new candidate reference genes and 8 commonly used reference genes in RNA-Seq datasets were compared. As shown in Fig 1 and Table 2, the 7 new candidate reference genes exhibited less variation in their expression levels in general.

**Table 1:** RT-qPCR primer sets used in this study and their target genes.

Gene name	Gene ID	Primer name	Forward primer (5’-3’)	Reverse primer (5’-3’)
*Bic-C1*	Glyma.08G349900	*Bic-C1*	GTATGCTGATCGGGTGGAGA	AGCTGGGGTTCATCATCCTC
*Bic-C2*	Glyma.03G064800	*Bic-C2*	GCCTTAATGATGTGAATGGT	AGAGATCATGTTCCCACTTG
*F-box protein2*	Glyma.02G273700	*F-box protein2*	TGAGAAAGCTGTTGAGGATT	GATTGCTCTTAAATCCATGC
*GPX*	Glyma.08G013800	*GPX*	TCTCTATTTCAGCCACTCGT	GGTTTGAAGGAAGTGTGAAG
*RHA*	Glyma.08G113000	*RHA*	ACTGGTAGATTTGCTGGAGA	CTTATTTGTGGCTCAAAACC
*SF*	Glyma.05G117600	*SF*	CTCTCTTCGACAAGTATGGG	CAAACTGGACCGTTATTTCG
*VPS-like*	Glyma.09G196600	*VPS-like*	AAAGAGTCTCATCCCACAAC	CGCATATTCCCAATCTCAGA
*ACT11* *	Glyma.18G290800; Glyma.02G091900	*ACT11*.*C*	ATTTTGACTGAGCGTGGTTATTCC	GCTGGTCCTGGCTGTCTCC
*ACT2/7* *	Glyma.19G147900	*ACT2/7*.*A*	CTTCCCTCAGCACCTTCCAA	GGTCCAGCTTTCACACTCCAT
*CYP* *	Glyma.12G024700	*CYP*.*B*	ACGACGAAGACGGAGTGG	CGACGACGACAGGCTTGG
*ELF1a* *	Glyma.05G114900; Glyma.19G052400	*ELF1a*	GACCTTCTTCGTTTCTCGCA	CGAACCTCTCAATCACACGC
*ELF1b* *	Glyma.02G276600; Glyma.14G039100	*ELF1b*.*B*	CCACTGCTGAAGAAGATGATGATG	AAGGACAGAAGACTTGCCACTC
*G6PD* *	Glyma.16G063200	*G6PD*	ACTCCTTGATACCGTTGTCCAT	GTTTGTTATCCGCCTACAGCCT
*TUB4* *	Glyma.03G124400; Glyma.19G127700	*TUB4*.*B*	TGGCGTCCACATTCATTG	GAACTCCATCTCGTCCAT
*UBQ10* *	Glyma.07G199900	*UBQ10*	TCCCACCAGACCAGCAGAG	CACGAAGACGCAACACAAGG

* Commonly used reference genes.

**Table 2:** Evaluation of selected reference genes using RNA-Seq data.

		Group 1			Group 2		
Gene name	Gene ID	Coefficient of Variation	Shapiro test p-value	KS-test p-value	Coefficient of Variation	Shapiro test p-value	KS-test p-value
***F-box protein2***	Glyma.02G273700	0.094	0.819	0.992	0.197	0.960	0.981
***RHA***	Glyma.08G113000	0.158	0.588	0.896	0.198	0.974	0.995
***Bic-C1***	Glyma.08G349900	0.106	0.390	0.747	0.152	0.026	0.460
***Bic-C2***	Glyma.03G064800	0.193	0.443	0.942	0.177	0.883	0.995
***VPS-like***	Glyma.09G196600	0.137	0.754	0.938	0.183	0.697	0.995
***SF***	Glyma.05G117600	0.181	0.140	0.663	0.137	0.081	0.760
***GPX***	Glyma.08G013800	0.161	0.924	0.994	0.236	0.187	0.813
***G6PD*** *	Glyma.16G063200	0.805	0.096	0.573	1.269	1.228E-05	0.130
***ACT2/7*** *	Glyma.19G147900	0.294	0.507	0.848	0.776	1.588E-05	0.096
***UBQ10*** *	Glyma.07G199900	0.652	0.087	0.389	0.856	2.837E-04	0.119
***TUB4*** *	Glyma.03G124400	0.459	0.003	0.369	0.730	0.001	0.301
***TUB4*** *	Glyma.19G127700	0.271	0.041	0.581	0.528	0.177	0.629
***ACT11*** *	Glyma.18G290800	0.315	0.999	0.999	0.730	0.000	0.152
***ACT11*** *	Glyma.02G091900	0.447	0.563	0.931	0.597	0.001	0.133
***ELF-1b*** *	Glyma.14G039100	0.382	0.061	0.732	0.555	0.001	0.223
***ELF-1b*** *	Glyma.02G276600	0.460	0.133	0.734	0.423	0.320	0.725
***ELF-1a*** *	Glyma.05G114900	0.140	0.609	0.876	0.414	0.004	0.488
***ELF-1a*** *	Glyma.19G052400	0.159	0.387	0.900	0.340	0.027	0.466
***CYP*** *	Glyma.12G024700	0.278	0.210	0.582	0.361	0.618	0.929

* Commonly used reference genes.

### Experimental validation using RT-qPCR

**Fig 2:**
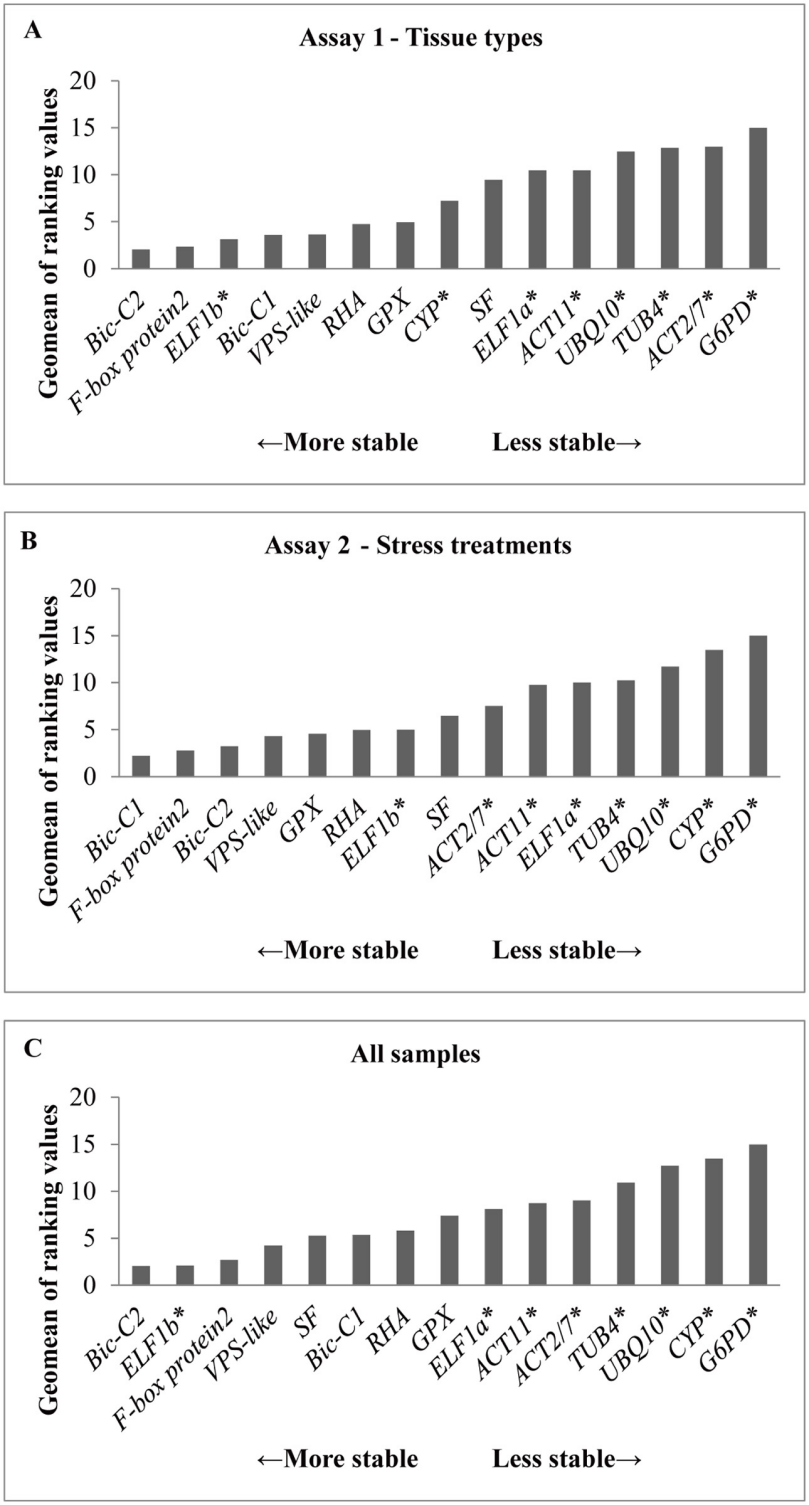
Geomeans of ranking values of (A) tissue types, (B) stress treatments, and (C) all samples. Genes are arranged in descending order of stability from left to right. Commonly used reference genes are marked with asterisk (*).

The primer sets of the 7 candidate reference genes were subjected to *in silico* PCR to ensure the specificity of the primers. Together with the primer sets for the 8 commonly used reference genes, two sets of experimental validation tests (a total of 26 samples) were performed (Table 3). To evaluate gene expression stability, we have adopted the RefFinder algorithm (http://www.leonxie.com/referencegene.php), which has integrated 4 commonly used stability evaluation programs: the comparative delta-Ct method, BestKeeper, Normfinder and geNorm, to generate a comprehensive ranking by calculating the geometric mean (geomean). The geomean and the stability ranking of the target genes are shown in Fig 2 and Table 4, respectively.

**Table 3:** Evaluation of reference genes using RT-qPCR assays.

Gene name^a^	Assay 1		Assay 2		All samples	
	Mean Ct	Mean ΔCt	Mean Ct	Mean ΔCt	Mean Ct	Mean ΔCt
***VPS-like***	28.55	0.85	28.92	1.22	28.84	1.13
***F-box protein2***	29.37	1.08	29.48	1.20	29.46	1.17
***SF***	29.76	1.06	29.98	1.28	29.93	1.23
***GPX***	26.05	1.75	25.40	1.11	25.55	1.25
***Bic-C1***	29.48	0.36	30.77	1.65	30.47	1.35
***RHA***	28.50	0.44	29.72	1.66	29.43	1.38
***Bic-C2***	28.94	0.87	29.83	1.75	29.62	1.55
***ELF1b*** *	23.01	0.99	23.80	1.78	23.62	1.60
***ELF1a*** *	23.36	1.49	23.58	1.71	23.53	1.66
***ACT11*** *	28.98	1.20	29.59	1.81	29.45	1.67
***CYP*** *	24.13	1.96	23.99	1.82	24.02	1.85
***ACT2/7*** *	24.88	1.11	25.96	2.19	25.71	1.94
***UBQ10*** *	24.18	2.65	23.32	1.80	23.52	1.99
***TUB4*** *	24.06	1.08	25.38	2.40	25.08	2.09
***G6PD*** *	26.28	3.39	26.37	3.48	26.35	3.46

^a^Assay 1: Tissue types; Assay 2: Stress treatments; All samples: all samples from assay 1 and assay 2.

* Commonly used reference genes,

**Table 4:** Ranking of reference genes based on the geomean of stability values.

**Stability ranking**	**Assay 1**	**Assay 2**	**All**
**1**	*Bic-C2*	*Bic-C1*	*Bic-C2*
**2**	*F-box protein2*	*F-box protein2*	*ELF1b* *
**3**	*ELF1b* *	*Bic-C2*	*F-box protein2*
**4**	*Bic-C1*	*VPS-like*	*VPS-like*
**5**	*VPS-like*	*GPX*	*SF*
**6**	*RHA*	*RHA*	*Bic-C1*
**7**	*GPX*	*ELF1b* *	*RHA*
**8**	*CYP* *	*SF*	*GPX*
**9**	*SF*	*ACT2/7* *	*ELF1a* *
**10**	*ELF1a* *	*ACT11* *	*ACT11* *
**11**	*ACT11* *	*ELF1a* *	*ACT2/7* *
**12**	*UBQ10* *	*TUB4* *	*TUB4* *
**13**	*TUB4* *	*UBQ10* *	*UBQ10* *
**14**	*ACT2/7* *	*CYP* *	*CYP* *
**15**	*G6PD* *	*G6PD* *	*G6PD* *

* Commonly used reference genes.

In validation assay 1 (tissue types), the expression of genes were investigated among different tissues including root, primary leaf, and trifoliate at the same growth stage in one wild and one cultivated soybean accession. The mean of two biological replicates was taken to represent a total of 6 samples. The 4 most stable reference genes were *Bic-C2*, *F-box protein 2*, *ELF1b*, and *Bic-C1*, of which the first two genes were new candidate reference genes. All candidate reference genes exhibited higher stability in expression than most of the commonly used reference genes except *ELF1b* (Fig 2A; Table 4).

In validation assay 2 (stress treatments), one wild and one cultivated soybean accession were subjected to near-isotonic NaCl or PEG treatment. Root and leaf samples were collected from untreated plants, as well as NaCl- or PEG-treated plants at 4 h and 24 h post-treatment. The mean of two biological replicates was taken to represent a total of 20 samples. Among the 15 primer sets that we tested, the majority of newly identified candidate reference genes ranked higher than the commonly used reference genes, e.g. *Bic-C1*, *F-box protein 2*, *Bic-C2*, and *VPS-like* were the 4 most stable reference genes (Fig 2B; Table 4).

To examine the overall stability of these 15 genes, the Ct values of all samples were pooled together to determine the best performing genes in RT-qPCR analyses (Table 3). The 7 candidate reference genes were ranked among the top 8, with *Bic-C2*, *ELF 1b*, *F-box protein2*, and *VPS-like* ranked the top four. On the other hand, *G6PD*, a commonly used reference gene, was found to be the least stable. The stability ranking is shown in Fig 2C and Table 4; the individual scores for comparative delta-Ct method, BestKeeper, NormFinder, and GeNorm were shown in S1 Fig.

## Discussion

To facilitate the analyses of soybean gene expressions using RT-qPCR, we evaluated previously reported reference genes and identified new candidate reference genes in this study. Due to the recent update of both the genomic sequence and the annotation of *Glycine max* William 82, the information on previously reported reference genes needs to be re-examined. We have reviewed 37 primer sets corresponding to 28 genes. *In silico* PCR analyses were performed to reveal the latest annotations and loci of the reported reference genes (S1 Table). Thirteen primer sets were found to target to multiple genes, with 4 of them giving multiple amplicons of different sizes, which may skew the RT-qPCR results under certain conditions [10, 22]. To further characterize the reported reference genes, the data of 26 RNA-Seq libraries were analyzed. The RNA-Seq data revealed that the previously reported reference genes may exhibit expression variations under different conditions. This result prompted us to search for new and improved candidate reference genes.

Although global normalization methods such as quartile normalization is commonly applied prior to differential gene expression analyses, the focus of this study is to identify candidate reference genes that exhibit a consistent expression pattern and therefore the fluctuation should be able to be modeled by a normal distribution. Two factors were considered: (1) the CoV; and (2) the p-value in the normality tests. The stringent CoV cut-off is to identify candidate genes with minimal variations in gene expression, which is similar to the approach previously adopted to find house-keeping exons using the data from the Human BodyMap Project [4]. It is also important to further prioritize the genes based on the nature of expression variations across samples. The two normality tests—Shapiro test and KS-test could determine whether the variations in gene expression follow a normal distribution, or whether they are skewed in certain samples or under certain conditions.

By considering both the CoV and the p-value of normality tests, 7 candidate reference genes were selected. They were subjected to two validation tests using RT-qPCR, together with 8 commonly used reference genes. Among the reported reference genes chosen, *G6PD* is the most unstable in the RT-qPCR validation tests, despite that it has been frequently used as a reference gene [10, 16]. Since the root is used for water absorption and the leaf for photosynthesis, glucose metabolism-related genes such as *G6PD* may thus be an inappropriate choice for normalization when comparing gene expressions in different tissues. This explains the relative instability of *G6PD* expression in the tissue-specific assays. Since sugar metabolism is also affected under abiotic stress [23], the expression of *G6PD* may also vary under environmental stress. It was also shown, by western blot analysis, that the expression of *G6PD* in soybean cultivars is induced under PEG 6000 treatment [24]. The instability of cytoskeleton-related genes such as *ACT2/7* and *ACT11* under stress treatments is probably related to the changes in cell morphology under water-related stress [25]. This finding is in agreement with a previous study which suggested that tubulin and actin are not desirable housekeeping genes for salt stress studies [26].

*ELF1b* gave the best performance among the 8 commonly used reference genes that were tested experimentally. However, while it has a high ranking in validation assay 1 (tissue types), its ranking in validation assay 2 (stress treatments) is mediocre. It is consistent with previous findings that *ELF1b* was the most stably expressed gene across different soybean tissues [10], while the expression of *ELF1b* under stress versus control conditions could be significantly different [11].

The candidate reference genes we identified here were solely based on an empirical analysis of the RNA-Seq data. To prevent unnecessary complications, each primer set we designed would only amplify a single target. All 7 candidate reference genes we selected for the RT-qPCR validation tests exhibited consistent expression levels, whereas *Bic-C2*, *F-box protein2*, and *VPS-like* gave the best overall performance (Tables 3 and 4).

To conclude, by utilizing a probabilistic approach in selecting normalization and reference genes, we identified 3 new and more appropriate reference genes for soybean gene expression studies. It is expected that with more soybean RNA-Seq data available from different growth stages and treatments, more comprehensive analyses could be performed, and a stable reference gene set across most conditions could be identified using the same methodology employed in this study.

## Materials and Methods

### RNA-Seq datasets and analyses

Soybean seeds from one cultivated soybean accession C08 and one wild soybean accession W05 [27] were germinated in the greenhouse on vermiculite in the dark for 72 hours. Seven-day-old seedlings were transferred to a hydroponic system with one-strength Hoagland’s solution. When the 1^st^ trifoliate was fully opened, samples of trifoliate leaves, primary leaves, and roots were collected, immediately frozen in liquid nitrogen, and stored at -80°C before RNA extraction. Total RNA was extracted and converted to cDNA (see below), followed by high-throughput sequencing using the Illumina Solexa GAII platform. A total of 6 paired-end sequencing libraries with a read length of 75bp and an insert size of 200bp were generated. The RNA-Seq data of W05 was submitted to the NCBI Sequence Read Archive (SRA) (SRR1185321 to SRR1185323) previously as part of the tool for the annotation of the W05 genome [28]. The RNA-Seq data of C08 has also been deposited to the NCBI SRA (SRR1737745 to SRR1737747). The raw reads were then subjected to quality checking by using Trimmomatic [29] with default parameters. Novoalign v3.02.07 (Novocraft Technologies Sdn Bhd, Malaysia) was used to perform reference assembly to the genome *Glycine max* 275 v2.0. The alignment statistics were shown in S2 Table.

Two additional RNA-Seq datasets were obtained from public databases. One of the datasets published in 2010 was obtained from SoyKB [19, 20]. The sequencing libraries—young leaf, seed (14–17 DAF), seed (10–13 DAF), seed (21 DAF), one cm pod, pod-shell (14–17 DAF), pod-shell (10–13 DAF), nodule, root, seed (25 DAF), seed (28 DAF), seed (35 DAF), and seed (42 DAF) were aligned to the reference genome *Glycine max* 275 v2.0 using Novoalign v3.02.07. The alignment statistics were shown in S2 Table. Another dataset published in Phytozome [21] containing the sequencing libraries of flower, leaf, nodule, root, root hair, shoot apical meristem, and stem was pre-aligned and downloaded as BAM files. The alignment statistics were shown in S2 Table. The expression of genes were measured by the number of mapped reads to the regions of coding sequences on the genome, with reference to the annotation of *Glycine max* William 82 275 v2.0.

### In silico PCR

For previously reported reference genes, a total of 37 primer sets were retrieved from 8 published reports [8, 10–16]. For candidate reference genes identified in this study, the primer sets were designed as described below. *In silico* PCR was performed using the BioPerl module—Bio::PrimerDesigner::ispcr (http://search.cpan.org/~smckay/Bio-PrimerDesigner-0.05/lib/Bio/PrimerDesigner/ispcr.pm) with the primer sets and *Glycine max* 275 v2.0 transcripts [17] as the inputs.

### Analyses of RNA-Seq data of 26 sequencing libraries

The coefficient of variation (CoV) of gene expression and the p-values of Shapiro-Wilk normality test (Shapiro test) and Kolmogorov-Smirnov test (KS test) for 25,036 genes were determined. For Group 1, the selected genes must (i) have a CoV < 0.2; and (ii) either the p-value of Shapiro test or KS test is larger than 0.6. For Group 2, the selected genes must (i) have a CoV < 0.24; and (ii) either the p-value of Shapiro test or KS test is larger than 0.45. The final selected genes must fulfill all the conditions listed in both Group 1 and Group 2. Under this stringent filtering criterion, only 136 out of 25,036 genes were shortlisted, and 7 of them were selected for experimental validation.

### Normalization with angular-based linear regression

The angular-based linear regression method assumes that in a pool of samples, there is a set of stably expressed genes across all samples. Assuming the vector of gene ***i*** is ${\vec{v}}_{i}$, which is constructed by the expressions ***v*** of this gene in all samples, the least angle regression minimizes the sum of angles between gene vectors ${\vec{v}}_{i}$ and reference vector $\vec{r}$, which is the unit vector representing the regression line. The regression line can be approximated by this equation:

$$\text{The}\;\text{sum}\;\text{of}\;\text{angles}\;\sim \;L\;=\;\sum_{i\;=\;1}^{n}\frac{\vec{r}\cdot {\vec{v}}_{i}}{\left|{\vec{v}}_{i}\right|}$$

where

$$\vec{r}\;=\;\left[\begin{array}{c} r_{1}\\ \begin{array}{c} r_{2}\\ \vdots \end{array}\\ r_{m} \end{array}\right]$$

$$\sum_{i\;=\;1}^{m}r_{i}^{2}\;=\;1$$

The regression line, or the regression vector, generates the maximum value of the function ***L***. To find the extreme values, the method of Lagrange multipliers is applied:

$$⋀\left(r_{1},\;r_{2}\ldots r_{m},\;\lambda \right)\;=\;L(r_{1},\;r_{2}\ldots r_{m})+\;\lambda (r_{1}^{2}+r_{2}^{2}+\ldots +\;r_{m}^{2}-1)$$

The partial derivatives are set to zero to find the extreme values:

From sample 1 to sample *m*:

$$\frac{\partial \Lambda }{\partial r_{i}}\;=\;0$$

And:

$$\frac{\partial \Lambda }{\partial \lambda }\;=\;0$$

There is only one extreme value in the function ***L***, which maximizes the function under given conditions. The size factor of sample ***k*** can thus be calculated by:

$$s_{k}\;=\;\frac{R_{k}}{\left|\vec{R}\right|}\cdot \sqrt{m}$$

Where

$$R_{k}\;=\;\sum_{i\;=\;1}^{n}\frac{v_{ik}}{\left|{\vec{v}}_{i}\right|},\;where\;k\;\in \left[1,\;m\right]$$

After getting the size factors, the normalization can be achieved by a single step:

$$Normalized\;expression\;=\;\frac{Raw\;expression}{Size\;factor}$$

### Finding the best regression model for angular-based linear regression

One way to find the best set of stably expressed genes is by using a probabilistic model based on Gaussian distribution. In this model, stably expressed genes are assumed to follow the Gaussian distribution, while unstably expressed genes are considered skewed and thus outliers. The best model has the largest likelihood.

Assuming that the normalized expression of gene ***i*** in sample ***k*** is ***W***_***ik***_, the normalized expressions should be re-scaled to ***u***_***ik***_ by the following calculation to set the equal weight to every gene:

$$u_{ik}\;=\;\frac{w_{ik}}{{\overset{-}{w}}_{i}\;}$$

where ${\overset{-}{w}}_{i}$ is the mean of the normalized expressions of gene ***i***. All gene expressions are then pooled. The mean ***μ*** and standard deviation ***σ*** are calculated by:

$$\;\mu \;=\;\frac{1}{m\times n}\cdot \sum_{i\;=\;1}^{n}\sum_{k\;=\;1}^{m}u_{ik}$$

$$\sigma \;=\;\sqrt{\frac{\sum_{i\;=\;1}^{n}\sum_{k\;=\;1}^{m}{\left(u_{ik}-\mu \right)}^{2}}{m\times n-1}}$$

Under the size factors calculated by the set of stably expressed genes, the likelihood can be calculated by:

$$Likelihood\;=\;\sum_{i\;=\;1}^{n}{log}_{10}(\prod_{k\;=\;1}^{m}P_{ik})\;$$

Where the probability ***P***_***ik***_ of gene ***i*** in sample ***k*** is:

$$P_{ik}\;=\;\{{}_{1-\Phi \left(-\left|\frac{u_{ik}-\mu }{\sigma }\right|\right)\times 2,\;if\;i\;is\;not\;a\;stably\;expressed\;gene}^{\Phi \left(-\left|\frac{u_{ik}-\mu }{\sigma }\right|\right)\times 2,\;if\;i\;is\;a\;stably\;expressed\;gene}$$

where **Φ** is the cumulative function of Gaussian distribution.

There are many methods to establish the best model with the maximum likelihood. One strategy is to first find the highest number of stably expressed genes, and then refine the combination of the stably expressed gene set around the number. In order to find the highest number of stably expressed genes, the initial size factors were calculated with the whole gene set. Then in each round, the gene with the smallest likelihood in the stably expressed gene set was eliminated, and the new initial size factors were calculated based on the new set of stably expressed genes, until all the genes were eliminated from the stably expressed gene set. The number of the stably expressed genes with the maximum likelihood during this process was selected. To further refine the stably expressed gene set, starting from the best gene set in the previous step, the state of the gene with the smallest probability was replaced by its opposite state, which means that if the gene is in the stably expressed gene set, it is eliminated and vice versa. The likelihood of the new set of stably expressed genes was then calculated. If the new likelihood was larger than the old one, the step would be repeated for the next gene in the list until the likelihood cannot be further increased.

### Sample preparation for RT-qPCR validation

For validation assay 1 (tissue types), samples were obtained using the same methodology as described for the RNA-Seq experiments above. For validation assay 2 (stress treatments), 7-day-old soybean seedlings (the wild accession W05 and the cultivated accession C08) were transferred to a hydroponic system with half-strength Hoagland’s solution. Near-isotonic salt or PEG treatment (150mM NaCl in half-strength Hoagland’s solution or 15% PEG 6000 in half-strength Hoagland’s solution) was applied when the primary leaves were fully opened [30]. Leaves and roots were harvested from untreated plants, and after treatment for 4 h and 24 h. Harvested samples were immediately frozen in liquid nitrogen and then stored at -80°C before RNA extraction. For each validation assay, biological repeats were performed.

Total RNAs were extracted from the ground samples by modified Phenol:chloroform:isoamylalcohol (25:24:1, v/v/v) protocol and subsequently purified with chloroform [31]. The purified RNA was quantified with a spectrophotometer and Qubit fluorometric RNA assay (Life technologies). The RNA concentration was adjusted to a preset concentration prior to DNase I (Life technologies) treatment. cDNA synthesis was carried out using SuperScript III Reverse Transcriptase (Life technologies) with the DNase-treated RNA, according to the manufacturer’s instruction. The resulting cDNAs were quantified with Qubit fluorometric ssDNA assay (Life technologies) and adjusted to 5ng/μl before being used as templates in RT-qPCR analyses.

### Design of primer sets

Primer sets for the 8 commonly used reference genes were adopted from previous reports (Table 1 and S1 Table). For new candidate reference genes identified in this study, the primers were designed to align in exon or exon-exon junction regions using the Primer 3 software and GenScript Real-time PCR Primer Design software, with the following parameters: (i) a single target by *in silico* PCR; (ii) a melting temperature of 55°C; (iii) primer lengths of 18–22 nt; and (iv) an amplicon size of 100-200bp.

### Quantitative PCR

All quantitative PCR (qPCR) reactions were performed in a CFX96 Touch Real-Time PCR system (Bio-rad) using SYBR green chemistry, with at least 3 technical repeats. The reaction mix per well was made up with 10μl iQ SYBR Green Supermix (Bio-rad), 0.3μl of forward/reverse primer (10μM), 3μl cDNA template, and 6.4μl Nuclease-free water. The thermal cycle was set according to the manufacturer’s protocol. At the end of each PCR run, a dissociation curve assay (from 95°C to 65°C) was carried out to verify the specificity of amplicons. The presence of single peak confirmed the specificity of amplicons.

### Statistical analyses for RT-qPCR results

The Ct value of each gene in each condition represents the mean of the Ct values of two biological replicates (each with 3 technical repeats). To compare the gene expression levels between samples under different conditions, the difference in Ct value between samples is calculated by ΔCt. ΔCt = Ct_sample_ − Ct_min_ (Ct_sample_ is the Ct value of the gene; Ct_min_ is the lowest Ct value of a gene; both were calculated by getting the mean of the Ct values of biological replicates within the sample set) [15].

Gene expression stability of our candidate reference genes and selected commonly used reference genes were evaluated using the comparative delta-Ct method [32], BestKeeper [33], Normfinder [34], and geNorm [7] algorithms via Ref-Finder (www.leonxie.com/referencegene.php). The new candidate reference genes and the commonly used reference genes were ranked based on the gene stability values calculated by each algorithm. A comprehensive geomean and stability ranking was obtained by the Ref-Finder, which incorporated results from the comparative delta-Ct method, BestKeeper, Normfinder, and geNorm algorithms.

## Supporting Information

S1 Fig Stability of gene expression. Samples were from different tissue types (A to D) and seedlings under stress treatments (E to H), calculated by the comparative delta-Ct method (A and E), BestKeeper (B and F), NormFinder (C and G), and geNorm (D and H) methods. From left to right: descending order of stability.
(DOCX)

S1 Table Summary of previously reported reference genes for soybean gene expression studies. (DOCX)

S2 Table Mapping statistics of 26 sequencing libraries. (DOCX)

S3 Table CoV and p-values of two normality tests of the 136 selected genes in both Group 1 and Group 2. (DOCX)
