# Supplementary material for: Using RNA-Seq Data to Evaluate Reference Genes Suitable for Gene Expression Studies in Soybean
Source: PLoS One. 2015 Sep 8;10(9):e0136343. doi: 10.1371/journal.pone.0136343 (PMC4562714; doi:10.1371/journal.pone.0136343)
Supplement: S1 Fig — Samples were from different tissue types (A to D) and seedlings under stress treatments (E to H), calculated by the comparative delta-Ct method (A and E), BestKeeper (B and F), NormFinder (C and G), and geNorm (D and H) methods. From left to right: descending order of stability. (DOCX) [file pone.0136343.s001.docx]

**S1 Fig.** Stability of gene expression. Samples were from different tissue types (A to D) and seedlings under stress treatments (E to H), calculated by the comparative delta-Ct method (A and E), BestKeeper (B and F), NormFinder (C and G), and geNorm (D and H) methods. From left to right: descending order of stability. * Commonly used reference gene.
