# Supplementary material for: Using RNA-Seq Data to Evaluate Reference Genes Suitable for Gene Expression Studies in Soybean
Source: PLoS One. 2015 Sep 8;10(9):e0136343. doi: 10.1371/journal.pone.0136343 (PMC4562714; doi:10.1371/journal.pone.0136343)
Supplement: S1 Table — (DOCX) [file pone.0136343.s002.docx]

**S1 Table. Summary of previously reported reference genes for soybean gene expression studies.**

| **Gene symbol** | ***In silico* PCR**  **amplicon**** | **Amplicon**  **size (bp)** | **Primer name** | **Forward primer (5'-3')** | **Reverse primer (5'-3')** | **Function** | **Refer-**  **ences** |
| --- | --- | --- | --- | --- | --- | --- | --- |
| ***ACT11**** | Glyma.18G290800 | 142 | ACT11.A | CGGTGGTTCTATCTTGGCATC | GTCTTTCGCTTCAATAACCCTA | Cytoskeletal protein | [1-4] |
|  | Glyma.18G290800;  Glyma.02G091900 | 126 | ACT11.B | ATCTTGACTGAGCGTGGTTATTCC | GCTGGTCCTGGCTGTCTCC |  | [5,6] |
|  | Glyma.18G290800;  Glyma.02G091900 | 126 | ACT11.C | ATTTTGACTGAGCGTGGTTATTCC* | GCTGGTCCTGGCTGTCTCC* |  | [7] |
| ***ACT2/7**** | Glyma.19G147900 | 119 | ACT2/7.A | CTTCCCTCAGCACCTTCCAA* | GGTCCAGCTTTCACACTCCAT* | Cytoskeletal protein | [1,3,7] |
|  | Glyma.04G215900;  Glyma.06G150100 | 91 | ACT2/7.B | AATTCACGAGACCACCTACAAC | TGAGCCACCACTAAGAACAATG |  | [5,6] |
| ***APC-like^Δ^*** | Glyma.18G036900 | 142 | APC-like | ATCATGAGTGAACAAAAAGGTTC | AGTCTGAAACAAACTCTCGCC | Anaphase promotion | [6] |
| ***CTL-like*** | Glyma.U017300 | 118 | CTL-like | TTGCTTGAGGGAGAAGATGAG | TTCCATATGCTGCAATCTGTAC | Plasma-  membrane choline transporter | [6] |
| ***CYP**** | Glyma.12G024700 | 154 | CYP.A | CGGGACCAGTGTGCTTCTTCA | CCCCTCCACTACAAAGGCTCG | Protein folding | [1-4] |
|  | Glyma.12G024700 | 130 | CYP.B | ACGACGAAGACGGAGTGG* | CGACGACGACAGGCTTGG* |  | [5-7] |
| ***ELF1a**** | Glyma.05G114900;  Glyma.19G052400 | 161 | ELF1a | GACCTTCTTCGT TTCTCGCA* | CGAACCTCTCAATCACACGC* | Translational elongation | [1,3,4,7] |
|  | Glyma.19G052400 | 1056 |  |  |  |  |  |
| ***ELF1b**** | Glyma.02G276600 | 102 | ELF1b.A | GTTGAAAAGCCAGGGGACA | TCTTACCCCTTGAGCGTGG | Translational elongation | [1-4] |
|  | Glyma.02G276600;  Glyma.14G039100 | 134 | ELF1b.B | CCACTGCTGAAGAAGATGATGATG* | AAGGACAGAAGACTTGCCACTC* |  | [5-7] |
| ***Glyma05g***  ***27480*** | Glyma.05G143100 | 103 | Glyma05g  27480 | CTTAAAAGACCTGTAGTTTGAGCA | ACACATTACTAAAGGGCCAAAG | Unknown | [6] |
| ***G6PD**** | Glyma.16G063200 | 126 | G6PD | ACTCCTTGATACCGTTGTCCAT* | GTTTGTTATCCGCCTACAGCCT* | Glucose metabolism | [1,4] |
| ***HDC*** | Glyma.08G050200 | 88 | HDC | AGGTCGTTGTTGTCTCAGGTG | CGTGCCGCTTCAGTCTCAG | Unwind DNA helix | [5] |
| ***IDE*** | Glyma.03G137100 | 78 | IDE | ATGAATGACGGTTCCCATGTA | GGCATTAAGGCAGCTCACTCT | Insulin-  degradation | [3] |
| ***MCD-like*** | Glyma.15G111500;  Glyma.09G007100 | 106,  111 | MCD-like | CCGAACACACATTCTTTCAGC | GATCGTTGGGCTTCATTCTG | Malonyl.CoA decarboxylation | [6] |
| ***MTP*** | Glyma.19G139800;  Glyma.03G137100 | 78 | MTP | CGCTCCAAGTGCTCCTCATTAG | TGAAGTAACCGACGCCAACG | Protein degradation | [5,6] |
| ***PEPKR1/ CDPK*** | Glyma.10G239600 | 68 | PEPKR1 | AGCAACCAAACAAATCCTGAACAAC | CCAACATCCAACTCTCCACAACC | Protein phosphorylation | [5] |
|  | Glyma.10G239600^#^ | N.A. (97bp^#^) | CDPK | TAAAGAGCACCATGCCTATCC | TGGTTATGTGAGCAGATGCAA |  | [2,3] |
| ***SKIP16*** | Glyma.12G051100 | 60 | SKIP16 | GAGCCCAAGACATTGCGAGAG | CGGAAGCGGAAGAACTGAACC | Protein binding | [5,6] |
| ***SUBI2*** | Glyma.13G117900^#^ | N.A. (84bp^#^) | SUBI2 | AGCTATTCGCAGTTCCCAAAT | CAGAGACGAACCTTGAGGAGA | Ubiquitin | [3] |
| ***TIP41*** | Glyma.20G130700 | 88 | TIP41 | AGGATGAACTCGCTGATAATGG | CAGAAACGCAACAGAAGAAACC | Signaling | [5] |
| ***TUA5*** | Glyma.05G157300 | 112 | TUA5.A | AGGTCGGAAACTCCTGCTGG | AAGGTGTTGAAGGCGTCGTG | Cytoskeletal protein | [1-4] |
|  | Glyma.05G157300;  Glyma.08G115100 | 103, 129 | TUA5.B | TGCCACCATCAAGACTAAGAGG | ACCACCAGGAACAACAGAAGG |  | [5-7] |
| ***TUA4*** | Glyma.20G136000 | 156 | TUA4 | CATACCCTAGAATCCATTTC | TGTACTTTCCGTGACGAG | Cytoskeletal protein | [7] |
| ***TUB4**** | Glyma.03G124400;  Glyma.19G127700 | 111 | TUB4.A | GGCGTCCACATTCATTGGA | CCGGTGTACCAATGCAAGAA | Cytoskeletal protein | [5,6] |
|  | Glyma.03G124400;  Glyma.19G127700 | 137 | TUB4.B | TGGCGTCCACATTCATTG* | GAACTCCATCTCGTCCAT* |  | [7] |
| ***UBC4*** | Glyma.18G216000 | 168; 241 | UBC4 | GAGCGAGCAGTTTCAGAC | CATAGGAGGGACGATACG | Ubiquitin conjugating enzyme | [4] |
| ***UBQ10**** | Glyma.07G199900 | 117 | UBQ10 | TCCCACCAGACCAGCAGAG* | CACGAAGACGCAACACAAGG* | Protein binding and modification | [5,6] |
| ***UKN1*** | Glyma.12G020500 | 74 | UKN1 | TGGTGCTGCCGCTATTTACTG | GGTGGAAGGAACTGCTAACAATC | Unknown | [5,6] |
| ***UKN2*** | Glyma.06G038500;  Glyma.04G037600 | 79 | UKN2.A | GCCTCTGGATACCTGCTCAAG | ACCTCCTCCTCAAACTCCTCTG | Unknown | [5,6] |
|  | Glyma.06G038500;  Glyma.04G037600 | 156 | UKN2.B | TGTGCTCTGTGAAGAGATTG | TCATAATCTGTGTGCAGTTC |  | [7] |
| ***YIF 1B-like*** | Glyma.08G256100 | 95 | YIF1B-like | CTGGGCAAAAAGAACTGAAT | AACTGAACTGACTAACAATTCGG | Integral membrane protein | [6] |
| ***40SRPS16*** | Glyma.02G103600 | 112 | 40SRPS16 | TCTGGGAATGTTGGAATTAAGC | CCTAGAATGCCTTAGCACATCC | 40s Ribosomal protein S16 | [6] |
| ***40SRPS10*** | Glyma.05G211100 | 82 | 40SRPS10 | TTCCACCTCGCAACCATGAT | CGAAGCAAACTCCCTCTTGG | 40s Ribosomal protein S10 | [6] |
| ***60s*** | Glyma.13G318800;  Glyma.12G182000 | 125 | 60s | AAAGTGGACCAAGGCATATCGTCG | TCAGGACATTCTCCGCAAGATTCC | 60s Ribosomal protein L30 | [3] |

* Commonly used reference genes validated by real-time RT-PCR in this study.

** *in silico* PCR was done with the primer sets and transcripts database of *Glycine max* genome assembly version 2.0, using the isPcr program.

***^Δ^*** *The APC-like gene exhibited very low read counts in all RNA-seq data and was not included in subsequent studies.*

# Previously reported reference genes that are predicted to have no amplicon using *in silico* PCR. The gene ID and amplicon sizes were provided in the corresponding literatures.

**Additional references**

1. Jian B, Liu B, Bi Y, Hou W, Wu C, et al. (2008) Validation of internal control for gene expression study in soybean by quantitative real-time PCR. BMC Mol Biol 9: 59.

2. Kulcheski FR, Marcelino-Guimaraes FC, Nepomuceno AL, Abdelnoor RV, Margis R (2010) The use of microRNAs as reference genes for quantitative polymerase chain reaction in soybean. Anal Biochem 406: 185-192.

3. Le DT, Aldrich DL, Valliyodan B, Watanabe Y, Ha CV, et al. (2012) Evaluation of candidate reference genes for normalization of quantitative RT-PCR in soybean tissues under various abiotic stress conditions. PLoS One 7: e46487.

4. Miranda Vde J, Coelho RR, Viana AA, de Oliveira Neto OB, Carneiro RM, et al. (2013) Validation of reference genes aiming accurate normalization of qPCR data in soybean upon nematode parasitism and insect attack. BMC Res Notes 6: 196.

5. Hu R, Fan C, Li H, Zhang Q, Fu YF (2009) Evaluation of putative reference genes for gene expression normalization in soybean by quantitative real-time RT-PCR. BMC Mol Biol 10: 93.

6. Li Q, Fan CM, Zhang XM, Fu YF (2012) Validation of reference genes for real-time quantitative PCR normalization in soybean developmental and germinating seeds. Plant Cell Rep 31: 1789-1798.

7. Ma S, Niu H, Liu C, Zhang J, Hou C, et al. (2013) Expression stabilities of candidate reference genes for RT-qPCR under different stress conditions in soybean. PLoS One 8: e75271.
