# Supplementary material for: Using RNA-Seq Data to Evaluate Reference Genes Suitable for Gene Expression Studies in Soybean
Source: PLoS One. 2015 Sep 8;10(9):e0136343. doi: 10.1371/journal.pone.0136343 (PMC4562714; doi:10.1371/journal.pone.0136343)
Supplement: S2 Table — (DOCX) [file pone.0136343.s003.docx]

**S2 Table. Mapping statistics of 26 sequencing libraries**

|  | Sample Identity | Paired Reads | Single Reads | % of Uniquely aligned reads |
| --- | --- | --- | --- | --- |
| Group 1 | Cultivar, Trifoliate | 16,475,011 | -- | 89.55 |
|  | Cultivar, Primary leaves | 17,198,072 | -- | 92.65 |
|  | Cultivar, Root tissue | 16,495,238 | -- | 89.91 |
|  | Wild, Trifoliate | 17,664,925 | -- | 89.90 |
|  | Wild, Primary leaves | 17,773,586 | -- | 87.84 |
|  | Wild, Root tissue | 17,727,652 | -- | 89.14 |
| Group 2 | 10A_nodule | -- | 8,655,757 | 67.77 |
|  | 2A_cm_pod | -- | 5,955,467 | 62.83 |
|  | 3A_-2_seed | -- | 6,534,652 | 47.05 |
|  | 4A_-2_shell | -- | 6,220,057 | 61.80 |
|  | 5A_-1_seed | -- | 5,661,899 | 45.94 |
|  | 6A_-1_shell | -- | 5,312,348 | 56.22 |
|  | 7A_0_seed | -- | 2,677,892 | 42.32 |
|  | 8A_young_leaf | -- | 6,576,692 | 69.99 |
|  | 9A_root | -- | 8,160,292 | 67.09 |
|  | soy_seed_A1 | -- | 2,873,808 | 49.16 |
|  | soy_seed_A2 | -- | 2,829,457 | 35.85 |
|  | soy_seed_A3 | -- | 5,281,772 | 65.12 |
|  | soy_seed_A4 | -- | 5,619,131 | 60.05 |
|  | Flower (BAM) | 83,608,846 | -- | 77.36 |
|  | Leaves (BAM) | 45,467,588 | -- | 75.70 |
|  | Nodules (BAM) | 44,075,984 | -- | 77.60 |
|  | Root (BAM) | 31,868,012 | -- | 75.89 |
|  | Root_hairs (BAM) | 53,447,007 | -- | 79.19 |
|  | Sam (BAM) | 41,117,258 | -- | 75.87 |
|  | Stem (BAM) | 45,043,424 | -- | 78.52 |
