# Supplementary material for: Using RNA-Seq Data to Evaluate Reference Genes Suitable for Gene Expression Studies in Soybean
Source: PLoS One. 2015 Sep 8;10(9):e0136343. doi: 10.1371/journal.pone.0136343 (PMC4562714; doi:10.1371/journal.pone.0136343)
Supplement: S3 Table — (DOCX) [file pone.0136343.s004.docx]

**S3 Table. CoV and p-values of two normality tests of the 136 selected genes in both Group 1 and Group 2**

| Gene | Group 1 - Coefficient of variation | Group 1 - Shapiro-Wilk normality test | Group 1 -Kolmogorov–Smirnov test | Group 2 - Coefficient of variation | Group 2 - Shapiro-Wilk normality test | Group 2 - Kolmogorov–Smirnov test |
| --- | --- | --- | --- | --- | --- | --- |
| Glyma.02G273700 | 0.094 | 0.819 | 0.992 | 0.197 | 0.960 | 0.981 |
| Glyma.08G113000 | 0.158 | 0.588 | 0.896 | 0.198 | 0.974 | 0.995 |
| Glyma.08G349900 | 0.106 | 0.390 | 0.747 | 0.152 | 0.026 | 0.460 |
| Glyma.03G064800 | 0.193 | 0.443 | 0.942 | 0.177 | 0.883 | 0.995 |
| Glyma.09G196600 | 0.137 | 0.754 | 0.938 | 0.183 | 0.697 | 0.995 |
| Glyma.14G053300 | 0.061 | 0.756 | 0.813 | 0.234 | 0.037 | 0.731 |
| Glyma.05G135800 | 0.063 | 0.367 | 0.739 | 0.184 | 0.886 | 0.909 |
| Glyma.10G291800 | 0.065 | 0.531 | 0.946 | 0.175 | 0.742 | 0.941 |
| Glyma.07G040900 | 0.068 | 0.469 | 0.960 | 0.202 | 0.268 | 0.588 |
| Glyma.19G153500 | 0.076 | 0.360 | 0.737 | 0.170 | 0.838 | 0.929 |
| Glyma.09G185000 | 0.090 | 0.865 | 0.988 | 0.221 | 0.728 | 0.931 |
| Glyma.05G227000 | 0.090 | 0.976 | 0.998 | 0.219 | 0.829 | 0.659 |
| Glyma.16G095200 | 0.095 | 0.428 | 0.970 | 0.236 | 0.910 | 0.999 |
| Glyma.09G188900 | 0.095 | 0.370 | 0.833 | 0.208 | 0.617 | 0.984 |
| Glyma.15G149100 | 0.096 | 0.171 | 0.650 | 0.184 | 0.575 | 0.516 |
| Glyma.19G049500 | 0.096 | 0.603 | 0.976 | 0.224 | 0.838 | 0.972 |
| Glyma.13G041500 | 0.099 | 0.371 | 0.713 | 0.226 | 0.872 | 0.968 |
| Glyma.06G014400 | 0.100 | 0.996 | 1.000 | 0.228 | 0.450 | 0.960 |
| Glyma.03G000600 | 0.100 | 0.542 | 0.956 | 0.216 | 0.944 | 0.996 |
| Glyma.19G160200 | 0.102 | 0.543 | 0.839 | 0.205 | 0.158 | 0.598 |
| Glyma.04G114800 | 0.102 | 0.855 | 0.988 | 0.200 | 0.778 | 0.700 |
| Glyma.11G211700 | 0.102 | 0.481 | 0.872 | 0.220 | 0.870 | 0.849 |
| Glyma.05G117600 | 0.181 | 0.140 | 0.663 | 0.137 | 0.081 | 0.760 |
| Glyma.08G013800 | 0.161 | 0.924 | 0.994 | 0.236 | 0.187 | 0.813 |
| Glyma.06G087700 | 0.107 | 0.822 | 0.989 | 0.228 | 0.894 | 0.991 |
| Glyma.10G172800 | 0.110 | 0.535 | 0.901 | 0.231 | 0.416 | 0.968 |
| Glyma.01G194600 | 0.110 | 0.828 | 0.978 | 0.185 | 0.634 | 0.819 |
| Glyma.04G193800 | 0.111 | 0.036 | 0.693 | 0.197 | 0.075 | 0.613 |
| Glyma.14G038900 | 0.111 | 0.422 | 0.987 | 0.228 | 0.406 | 0.944 |
| Glyma.17G037800 | 0.112 | 0.758 | 0.968 | 0.238 | 0.243 | 0.720 |
| Glyma.07G118300 | 0.115 | 0.157 | 0.689 | 0.213 | 0.946 | 0.991 |
| Glyma.15G274800 | 0.115 | 0.425 | 0.893 | 0.234 | 0.612 | 0.987 |
| Glyma.02G205500 | 0.115 | 0.570 | 0.905 | 0.229 | 0.730 | 0.871 |
| Glyma.13G087000 | 0.117 | 0.618 | 0.976 | 0.169 | 0.954 | 0.975 |
| Glyma.07G175000 | 0.118 | 0.276 | 0.709 | 0.224 | 0.873 | 0.813 |
| Glyma.02G186700 | 0.118 | 0.864 | 0.962 | 0.229 | 0.044 | 0.576 |
| Glyma.13G360400 | 0.119 | 0.569 | 0.813 | 0.205 | 0.150 | 0.786 |
| Glyma.03G191200 | 0.119 | 0.768 | 0.946 | 0.221 | 0.339 | 0.885 |
| Glyma.04G131700 | 0.120 | 0.585 | 0.816 | 0.218 | 0.304 | 0.926 |
| Glyma.06G140600 | 0.121 | 0.510 | 0.946 | 0.231 | 0.489 | 0.629 |
| Glyma.16G058300 | 0.122 | 0.791 | 0.918 | 0.222 | 0.026 | 0.774 |
| Glyma.14G166100 | 0.123 | 0.258 | 0.667 | 0.180 | 0.825 | 0.991 |
| Glyma.07G002500 | 0.125 | 0.656 | 0.849 | 0.239 | 0.655 | 0.975 |
| Glyma.05G154200 | 0.127 | 0.983 | 0.997 | 0.203 | 0.726 | 0.647 |
| Glyma.08G159600 | 0.130 | 0.066 | 0.638 | 0.230 | 0.515 | 0.867 |
| Glyma.17G027100 | 0.130 | 0.138 | 0.726 | 0.233 | 0.026 | 0.547 |
| Glyma.10G026200 | 0.132 | 0.624 | 0.869 | 0.211 | 0.342 | 0.724 |
| Glyma.19G091000 | 0.136 | 0.103 | 0.788 | 0.222 | 0.656 | 0.907 |
| Glyma.06G112400 | 0.138 | 0.072 | 0.757 | 0.236 | 0.531 | 0.597 |
| Glyma.10G178200 | 0.138 | 0.548 | 0.992 | 0.197 | 0.965 | 0.957 |
| Glyma.14G132600 | 0.140 | 0.454 | 0.913 | 0.233 | 0.276 | 0.709 |
| Glyma.16G155500 | 0.140 | 0.738 | 0.987 | 0.214 | 0.552 | 0.957 |
| Glyma.15G173200 | 0.142 | 0.649 | 0.915 | 0.227 | 0.967 | 0.994 |
| Glyma.13G003900 | 0.143 | 0.614 | 0.838 | 0.192 | 0.566 | 0.985 |
| Glyma.14G075000 | 0.143 | 0.446 | 0.937 | 0.226 | 0.312 | 0.700 |
| Glyma.02G097600 | 0.144 | 0.087 | 0.613 | 0.178 | 0.055 | 0.826 |
| Glyma.17G047600 | 0.144 | 0.504 | 0.669 | 0.233 | 0.026 | 0.496 |
| Glyma.07G232800 | 0.145 | 0.543 | 0.752 | 0.168 | 0.872 | 0.911 |
| Glyma.04G237000 | 0.145 | 0.351 | 0.890 | 0.232 | 0.619 | 0.901 |
| Glyma.05G089300 | 0.146 | 0.697 | 0.974 | 0.238 | 0.298 | 0.864 |
| Glyma.10G141500 | 0.147 | 0.962 | 0.998 | 0.225 | 0.492 | 0.808 |
| Glyma.13G112100 | 0.147 | 0.774 | 0.803 | 0.204 | 0.628 | 0.861 |
| Glyma.20G128800 | 0.150 | 0.014 | 0.611 | 0.224 | 0.718 | 0.741 |
| Glyma.11G004100 | 0.150 | 0.731 | 0.947 | 0.220 | 0.124 | 0.628 |
| Glyma.17G165100 | 0.151 | 0.921 | 0.980 | 0.200 | 0.091 | 0.598 |
| Glyma.07G244300 | 0.151 | 0.366 | 0.887 | 0.238 | 0.969 | 0.971 |
| Glyma.02G220800 | 0.152 | 0.192 | 0.681 | 0.224 | 0.524 | 0.864 |
| Glyma.12G223000 | 0.152 | 0.513 | 0.929 | 0.211 | 0.757 | 0.922 |
| Glyma.17G109500 | 0.157 | 0.570 | 0.793 | 0.210 | 0.808 | 0.912 |
| Glyma.19G015900 | 0.157 | 0.731 | 0.796 | 0.212 | 0.564 | 0.487 |
| Glyma.07G092900 | 0.158 | 0.997 | 0.999 | 0.223 | 0.980 | 1.000 |
| Glyma.14G126800 | 0.158 | 0.555 | 0.907 | 0.237 | 0.208 | 0.580 |
| Glyma.13G042100 | 0.159 | 0.478 | 0.967 | 0.201 | 0.394 | 0.481 |
| Glyma.20G133500 | 0.159 | 0.996 | 0.996 | 0.220 | 0.770 | 0.903 |
| Glyma.12G227900 | 0.160 | 0.119 | 0.662 | 0.201 | 0.298 | 0.884 |
| Glyma.08G291500 | 0.162 | 0.205 | 0.620 | 0.217 | 0.648 | 0.719 |
| Glyma.15G170700 | 0.167 | 0.604 | 0.878 | 0.239 | 0.804 | 0.967 |
| Glyma.19G067100 | 0.167 | 0.580 | 0.667 | 0.230 | 0.249 | 0.827 |
| Glyma.14G001200 | 0.169 | 0.520 | 0.963 | 0.215 | 0.253 | 0.603 |
| Glyma.14G123000 | 0.170 | 0.203 | 0.788 | 0.218 | 0.105 | 0.512 |
| Glyma.10G131700 | 0.171 | 0.969 | 0.987 | 0.237 | 0.024 | 0.719 |
| Glyma.08G227300 | 0.172 | 0.193 | 0.878 | 0.227 | 0.281 | 0.765 |
| Glyma.11G020200 | 0.172 | 0.494 | 0.962 | 0.211 | 0.747 | 0.841 |
| Glyma.03G136900 | 0.173 | 0.466 | 0.947 | 0.224 | 0.327 | 0.717 |
| Glyma.02G311300 | 0.173 | 0.685 | 0.981 | 0.189 | 0.768 | 0.928 |
| Glyma.04G253600 | 0.173 | 0.952 | 0.994 | 0.234 | 0.020 | 0.485 |
| Glyma.18G232400 | 0.175 | 0.512 | 0.929 | 0.231 | 0.413 | 0.849 |
| Glyma.05G004100 | 0.175 | 0.396 | 0.929 | 0.224 | 0.206 | 0.455 |
| Glyma.18G125700 | 0.176 | 0.848 | 0.976 | 0.144 | 0.672 | 0.926 |
| Glyma.02G212600 | 0.177 | 0.594 | 0.966 | 0.218 | 0.142 | 0.749 |
| Glyma.04G013900 | 0.177 | 0.901 | 0.992 | 0.190 | 0.121 | 0.505 |
| Glyma.09G044400 | 0.177 | 0.857 | 0.989 | 0.194 | 0.216 | 0.641 |
| Glyma.13G172200 | 0.178 | 0.777 | 0.935 | 0.146 | 0.398 | 0.923 |
| Glyma.08G048800 | 0.178 | 0.769 | 0.939 | 0.218 | 0.912 | 0.979 |
| Glyma.18G297400 | 0.179 | 0.500 | 0.929 | 0.220 | 0.491 | 0.599 |
| Glyma.08G053300 | 0.180 | 0.234 | 0.607 | 0.186 | 0.825 | 0.960 |
| Glyma.14G044300 | 0.181 | 0.422 | 0.958 | 0.219 | 0.201 | 0.689 |
| Glyma.03G107700 | 0.181 | 0.656 | 0.962 | 0.237 | 0.049 | 0.649 |
| Glyma.17G019800 | 0.182 | 0.077 | 0.643 | 0.213 | 0.520 | 0.765 |
| Glyma.06G108800 | 0.183 | 0.212 | 0.641 | 0.225 | 0.150 | 0.685 |
| Glyma.10G171500 | 0.183 | 0.311 | 0.826 | 0.238 | 0.717 | 0.898 |
| Glyma.01G053200 | 0.185 | 0.532 | 0.847 | 0.225 | 0.586 | 0.948 |
| Glyma.16G182300 | 0.185 | 0.623 | 0.833 | 0.197 | 0.756 | 0.969 |
| Glyma.02G270300 | 0.185 | 0.805 | 0.913 | 0.233 | 0.660 | 0.924 |
| Glyma.08G229400 | 0.186 | 0.297 | 0.775 | 0.235 | 0.389 | 0.953 |
| Glyma.09G215200 | 0.186 | 0.244 | 0.913 | 0.230 | 0.410 | 0.879 |
| Glyma.01G204000 | 0.187 | 0.226 | 0.676 | 0.212 | 0.310 | 0.912 |
| Glyma.12G226900 | 0.187 | 0.250 | 0.862 | 0.206 | 0.812 | 0.915 |
| Glyma.07G020100 | 0.187 | 0.055 | 0.621 | 0.231 | 0.745 | 0.944 |
| Glyma.06G074000 | 0.187 | 0.705 | 0.788 | 0.233 | 0.769 | 0.861 |
| Glyma.20G134100 | 0.187 | 0.987 | 1.000 | 0.198 | 0.649 | 0.837 |
| Glyma.02G044000 | 0.188 | 0.557 | 0.843 | 0.237 | 0.349 | 0.974 |
| Glyma.10G022000 | 0.188 | 0.249 | 0.921 | 0.153 | 0.154 | 0.507 |
| Glyma.18G019700 | 0.188 | 0.427 | 0.737 | 0.203 | 0.536 | 0.748 |
| Glyma.12G057300 | 0.189 | 0.689 | 0.865 | 0.234 | 0.091 | 0.559 |
| Glyma.10G000800 | 0.190 | 0.287 | 0.927 | 0.221 | 0.103 | 0.771 |
| Glyma.10G291300 | 0.190 | 0.809 | 0.992 | 0.239 | 0.842 | 0.970 |
| Glyma.06G324000 | 0.190 | 0.568 | 0.971 | 0.217 | 0.558 | 0.919 |
| Glyma.02G195600 | 0.192 | 0.106 | 0.754 | 0.233 | 0.434 | 0.544 |
| Glyma.04G074900 | 0.192 | 0.208 | 0.642 | 0.214 | 0.219 | 0.772 |
| Glyma.14G182400 | 0.193 | 0.442 | 0.800 | 0.146 | 0.978 | 0.973 |
| Glyma.17G233900 | 0.193 | 0.419 | 0.828 | 0.225 | 0.383 | 0.635 |
| Glyma.07G246900 | 0.193 | 0.935 | 0.936 | 0.216 | 0.090 | 0.752 |
| Glyma.15G126000 | 0.194 | 0.754 | 0.977 | 0.149 | 0.784 | 0.837 |
| Glyma.14G159000 | 0.194 | 0.963 | 0.990 | 0.173 | 0.670 | 0.884 |
| Glyma.17G129100 | 0.194 | 0.215 | 0.630 | 0.202 | 0.856 | 0.953 |
| Glyma.14G216900 | 0.195 | 0.452 | 0.828 | 0.218 | 0.963 | 0.994 |
| Glyma.10G106900 | 0.196 | 0.924 | 0.992 | 0.216 | 0.290 | 0.698 |
| Glyma.06G040600 | 0.196 | 0.463 | 0.908 | 0.190 | 0.468 | 0.891 |
| Glyma.12G182700 | 0.196 | 0.515 | 0.752 | 0.224 | 0.906 | 0.991 |
| Glyma.10G179900 | 0.196 | 0.683 | 0.863 | 0.230 | 0.844 | 0.935 |
| Glyma.19G096700 | 0.196 | 0.342 | 0.866 | 0.213 | 0.195 | 0.536 |
| Glyma.04G026300 | 0.198 | 0.669 | 0.978 | 0.190 | 0.345 | 0.709 |
| Glyma.05G185800 | 0.198 | 0.816 | 0.864 | 0.228 | 0.957 | 1.000 |
| Glyma.19G187900 | 0.198 | 0.940 | 0.999 | 0.226 | 0.719 | 0.845 |
| Glyma.13G040300 | 0.200 | 0.276 | 0.907 | 0.217 | 0.766 | 0.980 |
